# Genetic Variations in Metallothionein Genes and Susceptibility to Hypertensive Disorders of Pregnancy: A Case-Control Study

**DOI:** 10.3389/fgene.2022.830446

**Published:** 2022-06-06

**Authors:** Shudan Wei, Xiangyuan Yu, Xiaolan Wen, Min Zhang, Qi Lang, Ping Zhong, Bo Huang

**Affiliations:** ^1^ Guangxi Key Laboratory of Environmental Exposomics and Entire Lifecycle Health, Guangxi Health Commission Key Laboratory of Entire Lifecycle Health and Care, Guilin Medical University, Guilin, China; ^2^ Department of Obstetrics and Gynecology, The Second Affiliated Hospital of Guilin Medical University, Guilin, China

**Keywords:** hypertensive disorders of pregnancy, metallothionein, single-nucleotide polymorphism, case-control study, genetic susceptibility

## Abstract

**Background:** The involvement of oxidative stress in the pathological process of hypertensive disorders of pregnancy (HDP) gives rise to the interest in exploring the association of genetic variations in antioxidant metallothionein (*MT*) genes with HDP susceptibility.

**Methods:** Seventeen single-nucleotide polymorphisms(SNPs) in *MT* genes were selected to conduct genotyping based on a case-control study consisting of 371 HDP cases (pregnancy with chronic hypertension (66), gestational hypertension (172), and preeclampsia or preeclampsia superimposed on chronic hypertension (133)) and 479 controls. The association between SNPs in *MTs* and the risk of HDP was estimated with unconditional logistic regression analysis and further tested with the false-positive report probability (FPRP) procedure. The joint effects of SNPs on the HDP risk were assessed by haplotype analysis.

**Results:** After the adjustment for age and pre-pregnancy body mass index (pre-BMI) in the logistic regress analysis and followed by the FPRP test, the genetic variation rs10636 (OR = 0.46, 95% CI: 0.30–0.71 for GG vs. CC, *p* = 0.000 and OR = 0.48, 95% CI: 0.32–0.73 for GG vs. CG/CC, *p* = 0.001) in *MT2A* was associated with gestational hypertension. Other four SNPs, that is, rs11076161 (OR = 1.89, 95% CI: 1.35–2.63 for GG vs. GA/AA, *p* = 0.000) in *MT1A*; rs7191779 (OR = 1.54, 95% CI: 1.11–2.13 for CC vs. CG/GG, *p* = 0.010) in *MT1B*; rs8044719 (OR = 0.57, 95% CI: 0.40–0.80 for GT vs. GG, *p* = 0.001) in *MT1DP*; and rs8052334 (OR = 1.52, 95% CI: 1.10–2.11 for TT vs. TC/CC, *p* = 0.012) in *MT1B* were significantly associated with the susceptibility of HDP. The haplotype analysis among 11, 10, 10, and seven SNPs in *MT (MT1A*, *MT2A*, *MT1M*, *MT1B*, and *MT1DP)* genes showed that eight (A-C-G-T-C-G-A-G-C-G-C, OR = 4.559; A-C-T-C-C-C-A-G-C-G-C, OR = 5.777; A-C-T-T-C-G-A-G-C-G-C, OR = 4.590; G-A-T-C-C-G-C-G-G-C-C, OR = 4.065; G-A-T-C-G-C-C-G-G-C-C, OR = 4.652; G-A-T-T-C-C-C-G-G-C-C, OR = 0.404; G-C-T-C-C-C-A-G-G-C-C, OR = 1.901; G-C-T-T-C-C-A-G-G-C-C, and OR = 3.810), five (C-G-A-T-C-A-C-C-G-G, OR = 2.032; C-G-A-T-C-G-C-C-G-G, OR = 2.077; G-A-C-T-C-A-C-C-T-G, OR = 0.564; G-G-A-G-C-A-C-C-G-G, OR = 5.466; G-G-A-T-T-A-G-C-G-G, and OR = 0.284), five (A-C-G-T-C-G-A-G-C-C, OR = 2.399; A-C-T-C-C-C-C-T-G-G, OR = 0.259; G-A-T-C-C-C-C-G-G-C, OR = 1.572; G-A-T-C-G-C-C-G-G-C, OR = 0.001; G-C-T-C-G-C-A-G-G-C, and OR = 2.512), and five (A-C-T-C-C-C-G, OR = 0.634; G-A-G-C-C-C-G, OR = 4.047; G-A-T-T-G-C-G, OR = 0.499; G-C-G-T-C-A-G, and OR = 7.299; G-C-T-C-C-A-G, OR = 1.434) haplotypes were significantly associated with pregnancy with chronic hypertension, gestational hypertension, preeclampsia, or preeclampsia superimposed on chronic hypertension and HDP.

**Conclusion:** These variant *MT* alleles and their combination patterns may be used as genetic markers for predicting HDP susceptibility.

## Introduction

Hypertensive disorders of pregnancy (HDP) are unique diseases in obstetrics characterized by a systolic pressure equal to or above 140 mmHg and/or diastolic pressure equal to or above 90 mmHg (Wilkerson and Ogunbodede, 2019), which include pregnancy with chronic hypertension, gestational hypertension, preeclampsia (PE)/eclampsia, and chronic hypertension with superimposed PE/eclampsia (Khedagi and Bello, 2021). The average estimated worldwide prevalence of HDP are 5.2–8.2%, leading to 10–15% of maternal deaths, particularly in developing countries (Cao et al., 2020; Khan et al., 2006; Shah and Gupta, 2019). In China, the morbidity and mortality rates of HDP are up to 9.4% and ultimately 10.77–13.83% of maternal deaths, respectively (Cao et al., 2020; Lu et al., 2018). Although a variety of susceptibility factors of HDP have been identified, such as advanced maternal age, nulliparity, obesity, multiple gestations, and diabetes mellitus (Cao et al., 2020; Shah and Gupta, 2019; Su et al., 2020), the underlying mechanism of HDP remains unknown. However, oxidative stress has been proposed to be one of the leading pathophysiological processes of HDP (Phoswa and Khaliq, 2021). This occurs when the production of reactive oxygen species (ROS) and/or reactive nitrogen species (RNS) overwhelms the antioxidant capacity in the vascular walls, which causes constriction of blood vessels, thus resulting in the development of hypertension (Phoswa and Khaliq, 2021; Rozas-Villanueva et al., 2020; Sorriento et al., 2018).

Although exposures to environmental factors such as cadmium, noise, or traffic-related air pollution may increase the risk of HDP (Padula et al., 2021; Stanhope et al., 2021; Tanaka et al., 2019), HDP displays a genetic predisposition and some HDP-related susceptibility regions and genes have been identified (Umesawa and Kobashi, 2017). For instance, the genetic variations in endoplasmic reticulum aminopeptidase 1 and 2 (*ERAP1*, *2*) genes and deletion of the pregnancy-specific glycoprotein 11 (*PSG11*) gene are significantly associated with PE development (Ferreira et al., 2021; Seamon et al., 2020; Thakoordeen et al., 2018). A region in chromosome 2 predisposes Finland, Icelandic, Australian, and New Zealand populations to PE and, therefore, has been proposed as “PREG11” (preeclampsia, eclampsia gene 1) locus (Moses et al., 2000; Thakoordeen et al., 2018). A recent meta-analysis showed that rs3918242 in matrix metalloproteinase 9 (*MMP9*) genes was associated with the susceptibility of HDP, especially preeclampsia and gestational hypertension. The variant genotype and allele of rs3918242 C/T increased the risk of preeclampsia (OR = 1.48, 1.32; 95% CI: 1.18–1.86, 1.08–1.62) and gestational hypertension (OR = 2.23, 1.88; 95% CI: 1.52–3.28, 1.33–2.65) in the fixed-effect models (Zhou et al., 2021).

Metallothioneins (MTs) are a family of low–molecular weight (∼7,000 Da), highly conserved, and cysteine-rich metal-binding proteins (Shabb et al., 2017), which are involved in various physiological processes such as antioxidative stress, apoptosis, proliferation, angiogenesis, and detoxification of heavy metals (Bizon et al., 2017; Dutsch-Wicherek et al., 2008; Jin et al., 2002; Xue et al., 2012). In humans, MTs have four main subfamilies (MT1, MT2, MT3, and MT4) that are encoded by genes located at chromosome 16q13. *MT* genes comprise 12 functional isoforms (*MT1A*, *MT1B*, *MT1E*, *MT1F*, *MT1G*, *MT1H*, *MT1L*, *MT1M*, *MT1X*, *MT2A*, *MT3*, and *MT4*) and 7 nonfunctional alleles (*MT-1C*, *MT-1D*, *MT-1I*, *MT-1J*, *MT-1K*, *MT-1L*, and *MT-2B*) (McNeill et al., 2019; Raudenska et al., 2014). MTs can be highly induced by many different stimuli such as metals, hormones, cytokines, growth factors, oxidants, and irradiation (Alvarez-Barrios et al., 2021; Si and Lang, 2018) and thus have been proposed to be biomarkers for stress-related diseases/disorders including hypertension, tumors, diabetes mellitus, and metal poisoning (Sekovanic et al., 2020; Wang et al., 2018; Zhang et al., 2021; Zhang et al., 2020). In addition, MTs also function as powerful electrophilic scavengers and cytoprotective agents against oxidative stress and inflammatory injury by capturing ROS (Draganovic et al., 2016), and they keep protecting biological structures and the function of biomacromolecules from oxidative injuries (Jarosz et al., 2017).

HDP is directly associated with oxidative stress based on the fact that unsaturated lipids and thiol proteins in the membrane of endothelial cells in the vessel system are susceptible to be attacked by free radicals (Bizerea et al., 2018), and oxidative stress induces excessive production of ROS and RNS (Wisdom et al., 1991). This prompts us to systematically study the linkage of *MT* variations with the susceptibility of HDP. We conducted a case-control study to explore the association between genetic variations of *MT* genes (*MT1A*, *MT1B*, *MT1DP*, *MT1M*, *MT2A*, and *MT3*) and the risks of HDP by using the unconditional logistic regression, false-positive report probability (FPRP), and haplotype analysis in the study.

## Materials and Methods

### Study Population

A total of 371 HDP cases, including 66 of pregnancy with chronic hypertension (PCH), 172 of gestational hypertension (GH), and 133 of preeclampsia or preeclampsia superimposed on chronic hypertension (PE/PESCH), and 479 controls were recruited in the Affiliated Hospital of Guilin Medical University from September 2014 to June 2016 in this study. HDP was diagnosed by medical professionals in the hospital according to the recommendations from the International Society for the Study of Hypertension in Pregnancy (ISSHP) (Brown et al., 2018). The controls were matched to the cases by normal singleton pregnant women of ±3 years in the same period. The subjects with other basic diseases related to the liver, kidney, endocrine, nervous and respiratory systems, *etc.*, were excluded. Also, all participants were of Guangxi nationality who had lived in Guilin city for more than 10 years with no communication barriers. This study was approved by the Ethics Committee Review Boards of Guilin Medical University. Each participant signed the written informed consent form.

### Questionnaire Survey

A uniform epidemiological questionnaire was used to collect the demographic characteristics of the study populations, including age, height, weight, systolic blood pressure (SBP), diastolic blood pressure (DBP), personal history, and family history of hypertension and other diseases. The body mass index before gestation (pre-pregnancy BMI) was calculated as body weight (kg) divided by the square of height (m^2^). The biochemical data including fasting blood glucose (FBG), triglyceride (TG), total cholesterol (TC), and high-density lipoprotein cholesterol (HDL-c), which were determined during first trimester, were collected from medical records in hospitals.

### Blood Sample Collection and DNA Extraction

The peripheral blood sample (5 ml) was obtained from each participant, and genomic DNA was extracted by using a DNA extraction kit (Aidlab Biotechnologies Co., Ltd, China) and stored at −20°C for later analysis.

### SNP Selection

Seventeen functional SNPs of *MT* genes were chosen to conduct genotyping in the study according to the NIEHS SNPinfo Web Server (http://snpinfo.niehs.nih.gov/), NCBI dbSNP database (http://www.ncbi.nlm.nih.gov/projects/SNP), HapMap database (https://www.genome.gov/10001688), 1,000 Genomes (https://www.ncbi.nlm.nih.gov/variation/tools/1000genomes/), and the literature with the following criteria: 1) the SNPs are located at the regulatory region of *MT* genes, that is, the 5′ near gene, 5′ untranslated regions (UTR), 3′UTR, 3′near gene, splicing site (ESE or ESS), or miRNA binding site; 2) the minor allele frequency (MAF) of SNPs in Han Chinese in Beijing (CHB) or Asian/East Asian reported in HapMap or 1,000 Genomes is greater than 0.05; 3) the SNPs might affect transcription factor binding site (TFBS) activity in the putative promoter region or the posttranscriptional splicing process; 4) the linkage disequilibrium (LD) coefficient *r* ^2^ between candidate SNPs should be less than 0.8 (Rahimmadar et al., 2021; Xu et al., 2007) or one of them will be selected as tagSNP; and 5) the SNPs that have been identified to be related to *MT* functions by published studies even though they are located outside the functional regions will also be preferentially chosen.

A total of 191 SNPs were picked up in SNP Function Prediction by the gene name, in which 76 SNPs located out of functional regions and 70 SNPs with MAF<0.05 were excluded. The remaining 45 SNPs were maintained for LD analysis. Among them, six SNPs (rs11640851 (*MT1A*), rs8052394 (*MT1A*), rs8052334 (*MT1B*), rs1610216 (*MT2A*), rs28366003 (*MT2A*), and rs45570941 (*MT3*)) were included as tagSNPs (LD coefficient *r* ^2^ < 0.8); nine SNPs (rs1599823 (*MT1DP*), rs8044719 (*MT1DP*), rs9936741 (*MT1M*), rs1827208 (*MT1M*), rs10636 (*MT2A*), rs11644094 (*MT3*), rs8049883 (*MT1A*), rs11076161 (*MT1A*), and rs7196890 (*MT1A*)) were selected (LD coefficient *r* ^2^ > 0.8); and two SNPs (rs1580833 (*MT2A*) and rs7191779 (*MT1B*)) were taken from literatures (Supplementary Table S1).

### Genotyping

SNP genotyping was performed by using the Agena MassARRAY genotyping system (Agena; San Diego, CA) according to the manufacturer’s recommendation. The sequence of the SNP was obtained from the NCBI website, and the primers were designed and synthesized by Bio Miao Biological (Beijing, China). The main steps included the following: 1) polymerase chain reaction (PCR): each PCR master mix (5 μl) comprised 1 μl DNA template (10 ng/μl), 1.850 μl water, 0.625 μl of 1.25×PCR buffer (with 15 mmol/L MgCl_2_), 0.325 μl of 25 mmol/L MgCl_2_, 0.1 μl of 25 mmol/L dNTP mix, 1 μl of 0.5 μmol/L primer mix, and 0.1 μl of 5 U/μl Hot Star Taq polymerase. PCR was carried out at 94°C for 5 min; followed by running through 45 cycles consisting of 94°C for 20 s, 56°C for 30 s, and 72°C for 1 min; and finally incubated at 72°C for 3 min; 2) shrimp alkali enzyme purification (SAP) reaction: the volume contained 0.17 μl of 10 × SAP buffer, 0.3 μl of 1  U/μl SAP enzyme, and 1.53 μl water; 3) single-base extension reaction included 0.2 μl of iPLEX Buffer Plus, 0.2 μl of iPLEX termination mix, 0.94 μl of primer mix, 0.041 μl of iPLEX enzyme, 0.619 μl of water, and 7 μl of SAP + PCR reaction; and 4) resin purification. The data of the genotyping were analyzed with MassARRAY Typer software version 4.0.

### Statistical Analysis

The data were analyzed by using IBM SPSS Statistics 25. Student’s *t*-test was performed to analyze the quantitative data. The chi-square (χ^2^) test was applied to examine the accordance of genotypes of designated SNPs between HDP cases and controls. Hardy–Weinberg equilibrium (HWE) in the controls was tested by using a chi-square goodness-of-fit test. Unconditional logistic regression analysis was used to estimate the associations between the genotypes of SNPs and HDP susceptibility by computing the odds ratios (ORs) and their 95% confidence intervals (CIs). The statistical power of each SNP was estimated by using *post hoc* power analysis, in which the allele frequency and the effect (OR) of the SNP on the disease in studied populations were introduced into the logistic regression model. All statistical tests were two-tailed, and *p* < 0.05 was the criterion of statistical significance. In addition, FPRP analysis was used to further assess the robustness of the statistically significant associations. The threshold of the analysis was set to 0.2, and the prior probability was set to 0.1 to detect the efficacy for OR of 1.5 (risk) or 0.67 (protective), with an alpha level equal to the observed *p*-value (Wacholder et al., 2004; Zhu et al., 2013). LD and haplotype analysis were performed with the program SHEsis (http://27.223.74.230:8099/myAnalysis.php). The lowest frequency threshold was set as 0.01, and the haplotype with frequency less than 0.01 would be excluded from the analysis. The significance level (0.05/17 = 0.003) of Bonferroni’s correction was applied to account for multiple testing according to the number of designated SNPs in the study (Iskander et al., 2020; Yekutieli and Benjamini, 1999).

## Results

### The Characteristics of the Study Populations

The demographic characteristics of the study populations are listed in Table 1. There were no significant differences in age between HDP patients and controls (*p* > 0.05). However, the pre-pregnancy BMI (pre-BMI), systolic blood pressure (SBP), diastolic blood pressure (DBP), fasting blood glucose (FBG), triglyceride (TG), total cholesterol (TC), and high-density lipoprotein cholesterol (HDL-c) of HDP cases were higher than those of controls (*p* < 0.001).

**TABLE 1:** Characteristics of HDP cases and normotensive controls.

Characteristic	Case (n = 371)	Control (n = 479)	*P*
Age (year)	28.33 ± 4.72	28.77 ± 4.08	0.151
Pre-pregnancy BMI (pre-BMI)	26.32 ± 4.45	21.73 ± 3.19	**<0.001**
Systolic blood pressure (SBP)	133.82 ± 24.96	109.06 ± 9.77	**<0.001**
Diastolic blood pressure (DBP)	83.69 ± 17.25	68.76 ± 8.43	**<0.001**
Fasting blood glucose (FBG)	5.43 ± 1.60	4.41 ± 0.38	**<0.001**
Triglyceride (TG)	3.61 ± 1.72	2.40 ± 1.05	**<0.001**
Total cholesterol (TC)	6.57 ± 1.73	5.30 ± 1.08	**<0.001**
High-density lipoprotein cholesterol (HDL-c)	1.90 ± 0.46	1.66 ± 0.42	**<0.001**

Note: pre-BMI (kg/m^2^), SBP/DBP (mm/Hg), FBG (mmol/L), TG (mmol/L), TC (mmol/L), HDL-c (mmol/L).

Figures in bold type indicate statistical significance at the 0.05 level.

### Associations of SNPs in *MT* Genes With the Susceptibility of HDP Subtypes

Genotype distributions of the 17 selected SNPs in HDP and their subtypes, PCH, GH, and PE/PESCH, and controls are listed in Supplementary Table S2. All the genotype frequencies of the 17 SNPs agreed with the Hardy–Weinberg equilibrium in the study populations. In the logistic regression analysis, two, seven, and six SNPs were found to be significantly associated with PCH, GH, and PE/PESCH, respectively. After adjusting for age and pre-BMI, an increased risk of PCH was significantly associated with rs11076161 G variants in *MT1A*, and a decreased risk of PCH was associated with rs8044719 T variants in *MT1DP*. For GH, an increased risk was associated with rs11076161 G variants in *MT1A*, rs28366003 G variants in *MT2A*, and both of rs7191779 C and rs8052334 T variants in *MT1B*. A decreased risk of GH was significantly associated with rs10636 G variants in *MT2A*. For PE/PESCH, an increased risk was significantly associated with rs11076161 G variants in *MT1A* and rs11644094 G variants in *MT3*. Compared with individuals carrying the genotype CC of rs10636 in *MT2A*, those carrying the GC variants had a decreased risk, whereas the carriers with the GG variants of rs10636 had an increased risk of PE/PESCH compared with those carrying CG/CC alleles (Table 2).

**TABLE 2:** Association between assayed SNPs in *MT* genes and the susceptibility of PCH, GH, and PE/PESCH.

Group		Gene	SNP genotype	Call rate (%)	Case (n)/control (n)	*P* *^a^*	OR (95%CI)^b^	*P* *^b^*	OR (95%CI)^c^	*P* *^c^*
Pregnancy with chronic hypertension (PCH)	1	*MT1A*	rs11076161	99.4						
			AA		14/75	**0.035**	2.06 (1.01–4.23)	**0.048**	1.71 (0.77–3.79)	0.185
			GA		22/243		1.00		1.00	
			GG		29/159		2.02 (1.12–3.63)	**0.020**	2.05 (1.07–3.93)	**0.032**
			GA/GG vs. AA^d^		51/402	0.235	0.68 (0.36–1.29)	0.237	1.32 (1.23–1.42)	**0.000**
			GA/AA		36/318		1.00		1.00	
			GG^e^		29/159	0.073	1.61 (0.95–2.72)	0.075	1.33 (1.23–1.43)	**0.000**
	2	*MT1DP*	rs8044719	99.5						
			GG		56/344	0.054	1.00		1.00	
			GT		8/120		0.41 (0.19–0.88)	**0.023**	0.47 (0.21–1.06)	0.069
			TT		1/13		0.47 (0.06–3.68)	0.474	0.35 (0.04–3.16)	0.352
			GT/TT^d^		9/133	**0.016**	0.42 (0.20–0.86)	**0.019**	0.90 (0.84–0.97)	**0.004**
			GT/GG		64/464		1.00		1.00	
			TT^e^		1/13	1.000	0.56 (0.07–4.34)	0.577	0.40 (0.05–3.60)	0.416
Gestational hypertension (GH)	1	*MT2A*	rs10636	98.2						
			CC		9/22	**0.000**	0.74 (0.33–1.66)	0.460	0.62 (0.23–1.68)	0.350
			GC		94/169		1.00		1.00	
			GG		65/284		0.41 (0.28–0.60)	**0.000**	0.46 (0.30–0.71)	**0.000**
			GC/GG vs. CC^d^		159/453	0.706	0.86 (0.39–1.90)	0.706	1.09 (0.41–2.86)	0.869
			GG/CC		103/191		1.00		1.00	
			GG^e^		65/284	**0.000**	0.42 (0.30–0.61)	**0.000**	0.48 (0.32–0.73)	**0.001**
	2	*MT1A*	rs11076161	99.4						
			AA		16/75	**0.014**	1.00		1.00	
			GA		79/243		1.52 (0.84–2.77)	0.166	1.54 (0.78–3.04)	0.211
			GG		76/159		2.24 (1.22–4.10)	**0.009**	2.23 (1.12–4.44)	**0.023**
			GA/GG^d^		155/402	**0.040**	1.81 (1.02–3.20)	**0.042**	1.81 (0.95–3.47)	0.072
			GA/AA		95/318		1.00		1.00	
			GG^e^		76/159	**0.010**	1.60 (1.12–2.29)	**0.010**	1.58 (1.04–2.39)	**0.032**
	3	*MT2A*	rs28366003	99.8						
			AA		120/401	**0.000**	1.00		1.00	
			GA		45/70		2.15 (1.40–3.29)	**0.000**	1.84 (1.11–3.02)	**0.017**
			GG		7/8		2.92 (1.04–8.23)	**0.042**	2.68 (0.77–9.31)	0.121
			GA/GG^d^		52/78	**0.000**	2.23 (1.49–3.34)	**0.000**	1.91 (1.19–3.08)	**0.008**
			GA/AA		165/471		1.00		1.00	
			GG^e^		7/8	0.081	2.50 (0.89–7.00)	0.081	2.37 (0.69–8.21)	0.173
	4	*MT3*	rs45570941	99.3						
			GG		149/382	0.120	1.00		1.00	
			CG		21/87		0.62 (0.37–1.03)	0.067	0.71 (0.40–1.28)	0.251
			CC		1/7		0.37 (0.05–3.00)	0.349	0.66 (0.08–5.81)	0.707
			CG/CC^d^		22/94	**0.044**	0.60 (0.36–0.99)	**0.046**	0.71 (0.40–1.25)	0.233
			CG/GG		170/469		1.00		1.00	
			CC^e^		1/7	0.688	0.39 (0.05–3.23)	0.385	0.70 (0.08–6.12)	0.743
	5	*MT1B*	rs7191779	99.1						
			GG		15/65	**0.011**	1.00		1.00	
			CG		66/221		1.29 (0.69–2.42)	0.419	1.02 (0.50–2.09)	0.952
			CC		90/189		2.06 (1.12–3.82)	**0.021**	1.71 (0.85–3.45)	0.134
			CG/CC^d^		156/410	0.094	1.65 (0.91–2.98)	0.097	1.34 (0.68–2.63)	0.394
			CG/GG		81/286		1.00		1.00	
			CC^e^		90/189	**0.004**	1.68 (1.18–2.39)	**0.004**	1.68 (1.12–2.53)	0.013
	6	*MT1DP*	rs8044719	99.5						
			GG		139/344	0.081	1.00		1.00	
			GT		30/120		0.62 (0.40–0.97)	**0.035**	0.81 (0.49–1.34)	0.412
			TT		3/13		0.57 (0.16–2.04)	0.388	0.60 (0.15–2.39)	0.465
			GT/TT^d^		33/133	**0.025**	0.61 (0.40–0.94)	**0.026**	0.79 (0.48–1.27)	0.326
			GT/GG		169/464		1.00		1.00	
			TT^e^		3/13	0.579	0.63 (0.18–2.25)	0.480	0.62 (0.16–2.49)	0.503
	7	*MT1B*	rs8052334	98.9						
			CC		15/65	**0.011**	1.00		1.00	
			TC		66/224		1.28 (0.68–2.39)	0.443	1.00 (0.49–2.05)	0.996
			TT		89/188		2.05 (1.11–3.80)	**0.022**	1.68 (0.83–3.40)	0.148
			TC/TT^d^		155/412	0.102	1.63 (0.90–2.94)	0.105	1.31 (0.67–2.58)	0.431
			TC/CC		81/289		1.00		1.00	
			TT^e^		89/188	**0.003**	1.69 (1.19–2.40)	**0.004**	1.68 (1.12–2.54)	**0.013**
Preeclampsia or preeclampsia superimposed on chronic hypertension (PE/PESCH)	1	*MT2A*	rs10636	98.2						
			CC		13/22	**0.000**	1.00		1.00	
			GC		17/169		0.17 (0.07–0.40)	**0.000**	0.22 (0.08–0.60)	**0.003**
			GG		97/284		0.58 (0.28–1.19)	0.137	0.65 (0.27–1.58)	0.339
			GC/GG^d^		114/453	**0.017**	0.43 (0.21–0.87)	**0.019**	0.49 (0.20–1.18)	0.111
			GG/CC		30/191		1.00		1.00	
			GG^e^		97/284	**0.001**	2.18 (1.39–3.41)	**0.001**	2.03 (1.21–3.41)	**0.008**
	2	*MT1A*	rs11076161	99.4						
			AA		18/75	**0.003**	1.19 (0.65–2.17)	0.569	1.03 (0.51–2.09)	0.937
			GA		49/243		1.00		1.00	
			GG		65/159		2.03 (1.33–3.09)	**0.001**	1.95 (1.18–3.22)	**0.009**
			GA/GG vs. AA^d^		114/402	0.555	1.18 (0.68–2.06)	0.556	1.34 (0.69–2.59)	0.383
			GA/AA		67/318		1.00		1.00	
			GG^e^		65/159	**0.001**	1.94 (1.31–2.87)	**0.001**	1.94 (1.22–3.09)	**0.005**
	3	*MT3*	rs11644094	99.2						
			AA		85/357	0.068	1.00		1.00	
			AG		42/110		1.60 (1.05–2.46)	**0.030**	1.70 (1.00–2.89)	**0.050**
			GG		4/9		1.87 (0.56–6.21)	0.309	1.59 (0.35–7.22)	0.549
			AG/GG^d^		46/119	**0.021**	1.62 (1.07–2.46)	**0.022**	1.69 (1.01–2.83)	0.045
			AG/AA		127/467		1.00		1.00	
			GG^e^		4/9	0.492	1.63 (0.50–5.39)	0.420	1.40 (0.31–6.29)	0.660
	4	*MT1DP*	rs8044719	99.5						
			GG		109/344	**0.040**	1.00		1.00	
			GT		22/120		0.58 (0.35–0.96)	**0.033**	0.26 (0.03–2.21)	0.215
			TT		1/13		0.24 (0.03–1.88)	0.175	0.70 (0.39–1.25)	0.230
			GT/TT^d^		23/133	**0.015**	0.55 (0.33–0.89)	**0.016**	0.65 (0.37–1.14)	0.134
			GT/GG		131/464		1.00		1.00	
			TT^e^		1/13	0.322	0.27 (0.04–2.10)	0.212	0.28 (0.03–2.38)	0.241
	5	*MT1A*	rs8049883	99.4						
			AA		15/37	**0.045**	1.00		1.00	
			GA		36/183		0.49 (0.24–0.98)	**0.042**	0.63 (0.27–1.48)	0.290
			GG		81/256		0.78 (0.41–1.50)	0.455	1.04 (0.47–2.31)	0.921
			GA/GG^d^		117/439	0.192	0.66 (0.35–1.24)	0.194	0.87 (0.40–1.89)	0.721
			GA/AA		51/220		1.00		1.00	
			GG^e^		81/256	0.121	1.37 (0.92–2.02)	0.122	1.48 (0.93–2.38)	0.101
	6	*MT1B*	rs8052334	98.9						
			CC		19/65	0.077	1.39 (0.76–2.54)	0.279	1.32 (0.64–2.72)	0.449
			TC		47/224		1.00		1.00	
			TT		64/188		1.62 (1.06–2.48)	**0.025**	1.55 (0.94–2.55)	0.083
			TC/TT vs. CC^d^		111/412	0.772	0.92 (0.53–1.60)	0.772	0.95 (0.49–1.86)	0.880
			TC/CC		66/289		1.00		1.00	
			TT^e^		64/188	**0.044**	1.49 (1.01–2.20)	**0.045**	1.45 (0.91–2.30)	0.115

a Two-sided chi-square test for genotype distribution between cases and controls.

b Unadjusted for age and pre-pregnancy BMI in a logistic regression model.

c Adjusted for age and pre-pregnancy BMI in a logistic regression model.

d Tested for a dominant genetic model.

e Tested for a recessive genetic model.

The significance level of Bonferroni’s correction for multiple testing is 0.003.

Figures in bold type indicate statistical significance at the 0.05 level.

Italic values indicate the genes rather proteins. Italic P is the statistical convention.

To further test the associations estimated from the logistic regression analysis, FPRP analyses were conducted. Under a prior probability of 0.1, the OR for a specific genotype was estimated to be 0.67/1.5 (protection/susceptibility), and *p* < 0.2 was the criterion of statistical significance (Zhu et al., 2013). It turned out that the association of rs11076161 and rs8044719 with PCH and the association of rs10636 with GH remained (*p* < 0.2). However, the statistical powers of rs11076161 (GG vs. GA/AA) and rs8044719 (GT/TT vs. GG) related to PCH were only 0.180 and 0.052, respectively. Moreover, the association between SNPs in *MTs* and PE/PESCH disappeared (Table 4).

### Associations of SNPs in *MT* Genes With HDP Susceptibility

Since oxidative stress and hypertension are the common pathophysiological processes of HDP (Phoswa and Khaliq, 2021), we merged HDP subtypes to estimate the association of disease risks with SNPs in *MTs*. In the single-locus analyses, seven SNPs were found to be related to HDP. After adjustment for age and pre-BMI, four SNPs including rs11076161 (OR = 2.02, 95% CI: 1.22–3.36 for GG vs. AA and OR = 1.89, 95% CI: 1.35–2.63 for GG vs. GA/AA) in *MT1A*; rs7191779 (OR = 1.52, 95% CI: 1.08–2.15 for CC vs. CG and OR = 1.54, 95% CI: 1.11–2.13 for CC vs. CG/GG) in *MT1B*; rs8044719 (OR = 0.65, 95% CI: 0.44–0.95 for GT/TT vs. GG) in *MT1DP*; and rs8052334 (OR = 1.53, 95% CI: 1.08–2.16 for TT vs. TC and OR = 1.52, 95% CI: 1.10–2.11 for TT vs. TC/CC) in *MT1B* remained to be associated with HDP. Furthermore, the association of decreased HDP risk with rs8052394 (OR = 0.69, 95% CI: 0.49–0.98 for GA vs. AA, OR = 0.69, 95% CI: 0.50–0.96 for GA/GG vs. AA) in *MT1A* and an increased HDP risk with rs8049883 (OR = 1.50, 95% CI: 1.08–2.09 for GA/GG vs. GG) in *MT1A* emerged (Table 3).

**TABLE 3:** Association between assayed SNPs in *MT* genes and the susceptibility of HDP.

	Gene	SNP genotype	Call rate (%)	Case (n)/control (n)	*P* *^a^*	OR (95%CI)^b^	*P* *^b^*	OR (95%CI)^c^	*P* *^c^*
1	*MT2A*	rs10636	98.2						
		CC		29/22	0.110	1.00		1.00	
		GC		129/169		0.58 (0.32–1.06)	0.074	0.57 (0.28–1.16)	0.119
		GG		202/284		0.54 (0.30–0.97)	**0.038**	0.54 (0.27–1.07)	0.078
		GC/GG^d^		331/453	**0.041**	0.55 (0.31–0.98)	**0.043**	0.55 (0.28–1.08)	0.083
		GG/CC		158/191		1.00		1.00	
		GG^e^		202/284	0.286	0.86 (0.65–1.14)	0.286	0.87 (0.62–1.21)	0.410
2	*MT1A*	rs11076161	99.4						
		AA		48/75	**0.001**	1.00		1.00	
		GA		150/243		0.97 (0.64–1.46)	0.865	1.10 (0.67–1.81)	0.709
		GG		170/159		1.67 (1.10–2.55)	**0.017**	2.02 (1.22–3.36)	**0.006**
		GA/GG^d^		320/402	0.273	1.24 (0.84–1.84)	0.274	1.46 (0.92–2.34)	0.110
		GA/AA		198/318		1.00		1.00	
		GG^e^		170/159	**0.000**	1.72 (1.30–2.27)	**0.000**	1.89 (1.35–2.63)	**0.000**
3	*MT3*	rs45570941	99.3						
		GG		319/382	**0.041**	1.00		1.00	
		CG		44/87		0.61 (0.41–0.90)	**0.012**	0.72 (0.46–1.15)	0.171
		CC		5/7		0.86 (0.27–2.71)	0.791	1.14 (0.31–4.17)	0.840
		CG/CC^d^		49/94	**0.013**	0.62 (0.43–0.91)	**0.014**	0.76 (0.49–1.18)	0.215
		CG/GG		363/469		1.00		1.00	
		CC^e^		5/7	0.892	0.92 (0.29–2.93)	0.892	1.20 (0.33–4.38)	0.782
4	*MT1B*	rs7191779	99.1						
		GG		42/65	**0.041**	0.97 (0.63–1.51)	0.897	0.96 (0.56–1.63)	0.871
		CG		147/221		1.00		1.00	
		CC		178/189		1.42 (1.06–1.90)	**0.020**	1.52 (1.08–2.15)	**0.018**
		CG/CC vs. GG^d^		325/410	0.333	1.23 (0.81–1.86)	0.334	1.30 (0.79–2.14)	0.312
		CG/GG		189/286		1.00		1.00	
		CC^e^		178/189	**0.011**	1.43 (1.08–1.88)	**0.012**	1.54 (1.11–2.13)	**0.010**
5	*MT1DP*	rs8044719	99.5						
		GG		304/344	**0.002**	1.00		1.00	
		GT		60/120		0.57 (0.40–0.80)	**0.001**	0.68 (0.46–1.02)	0.061
		TT		5/13		0.44 (0.15–1.24)	0.118	0.39 (0.12–1.28)	0.120
		GT/TT^d^		65/133	**0.000**	0.55 (0.40–0.77)	**0.001**	0.65 (0.44–0.95)	**0.028**
		GT/GG		364/464		1.00		1.00	
		TT^e^		5/13	0.171	0.49 (0.17–1.39)	0.179	0.42 (0.13–1.38)	0.154
6	*MT1A*	rs8049883	99.4						
		AA		33/37	0.126	1.00		1.00	
		GA		117/183		0.72 (0.43–1.21)	0.213	0.80 (0.43–1.52)	0.499
		GG		219/256		0.96 (0.58–1.59)	0.871	1.26 (0.68–2.31)	0.462
		GA/GG^d^		336/439	0.541	0.86 (0.53–1.40)	0.541	1.06 (0.59–1.92)	0.840
		GA/AA		150/220		1.00		1.00	
		GG^e^		219/256	0.106	1.26 (0.95–1.65)	0.106	1.50 (1.08–2.09)	**0.017**
7	*MT1B*	rs8052334	98.9						
		CC		42/65	**0.035**	0.99 (0.64–1.54)	0.969	1.03 (0.60–1.74)	0.928
		TC		146/224		1.00		1.00	
		TT		176/188		1.44 (1.07–1.93)	**0.015**	1.53 (1.08–2.16)	**0.017**
		TC/TT vs. CC^d^		322/412	0.368	1.21 (0.80–1.83)	0.368	1.21 (0.73–2.00)	0.454
		TC/CC		188/289		1.00		1.00	
		TT^e^		176/188	**0.010**	1.44 (1.09–1.90)	**0.010**	1.52 (1.10–2.11)	**0.012**
8	*MT1A*	rs8052394	99.6						
		AA		216/255	0.222	1.00		1.00	
		GA		123/187		0.98 (0.59–1.65)	0.088	0.69 (0.49–0.98)	**0.037**
		GG		30/36		0.78 (0.58–1.04)	0.951	0.69 (0.37–1.31)	0.260
		GA/GG^d^		153/233	0.132	0.81 (0.62–1.07)	0.132	0.69 (0.50–0.96)	**0.028**
		GA/AA		339/442				1.00	
		GG^e^		30/36	0.747	1.09 (0.66–1.80)	0.747	0.80 (0.43–1.49)	0.484
9	*MT1M*	rs9936741	99.5						
		TT		310/367	**0.038**	1.00		1.00	
		CT		58/102		0.67 (0.47–0.96)	**0.029**	0.77 (0.51–1.17)	0.228
		CC		2/7		0.34 (0.07–1.64)	0.178	0.17 (0.02–1.22)	0.078
		CT/CC^d^		60/109	**0.016**	0.65 (0.46–0.92)	**0.016**	0.72 (0.48–1.09)	0.123
		CT/TT		368/469		1.00		1.00	
		CC^e^		2/7	0.332	0.36 (0.08–1.76)	0.209	0.17 (0.02–1.28)	0.086

a Two-sided chi-square test for genotype distribution between cases and controls.

b Unadjusted for age and pre-pregnancy BMI in a logistic regression model.

c Adjusted for age and pre-pregnancy BMI in a logistic regression model.

d Tested for a dominant genetic model.

e Tested for a recessive genetic model.

The significance level of Bonferroni’s correction for multiple testing is 0.003.

Figures in bold type indicate statistical significance at the 0.05 level.

Italic values indicate the genes rather proteins. Italic P is the statistical convention.

After the FPRP analyses, the four SNPs, that is, rs11076161, rs7191779, rs8044719, and rs8052334 had robust associations with HDP risks. In the heterozygous genotype comparison of rs8044719 (GT vs. GG), recessive models of rs11076161 (GG vs. GA/AA), rs7191779 (CC vs. CG/GG), and rs8052334 (TT vs. TC/CC), with their observed *p* values were 0.052, 0.013, 0.167, and 0.189, respectively (Table 4). Furthermore, the associations of HDP risks with rs8044719 and rs11076161 remained statistically significant following Bonferroni’s correction (Table 3).

**TABLE 4:** False-positive report probability (FPRP) analysis for the association between SNPs in *MT* genes and the susceptibility of PCH, GH, PE/PESCH, and HDP.

Group	SNP genotype	*P*	OR (95%CI)	Statistical power	Prior probability					
					0.25	0.1	0.01	0.001	0.0001	0.00001
Pregnancy with chronic hypertension (PCH)	rs11076161									
	AA vs. GA	0.048^a^	2.06 (1.01–4.23)	0.515	0.429	0.693	0.961	0.996	1.000	1.000
	GG vs. GA	0.032^b^	2.05 (1.07–3.93)	0.670	0.350	0.618	0.947	0.994	0.999	1.000
	GA/GG vs. AA	0.000^b^	1.32 (1.23–1.42)	0.126	0.000	**0.001**	0.010	0.091	0.500	0.909
	GG vs. GA/AA	0.000^b^	1.33 (1.23–1.43)	0.180	0.000	**0.001**	0.010	0.091	0.500	0.909
	rs8044719									
	GT vs. GG	0.023^a^	0.41 (0.19–0.88)	0.675	0.393	0.660	0.955	0.995	1.000	1.000
	GT/TT vs. GG	0.004^b^	0.90 (0.84–0.97)	0.052	0.012	**0.035**	0.284	0.800	0.976	0.998
Gestational hypertension (GH)	rs10636									
	GG vs. GC	0.000^b^	0.46 (0.30–0.71)	0.988	0.016	**0.046**	0.347	0.843	0.982	0.998
	GG vs. CG/CC	0.001^b^	0.48 (0.32–0.73)	0.984	0.037	**0.103**	0.559	0.928	0.992	0.999
	rs11076161									
	GG vs. AA	0.023^b^	2.23 (1.12–4.44)	0.770	0.344	0.612	0.945	0.994	0.999	1.000
	GA/GG vs. AA	0.042^a^	1.81 (1.02–3.20)	0.545	0.325	0.591	0.941	0.994	0.999	1.000
	GG vs. GA/AA	0.032^b^	1.58 (1.04–2.39)	0.702	0.189	0.412	0.885	0.987	0.999	1.000
	rs28366003									
	GA vs. AA	0.017^b^	1.84 (1.11–3.02)	0.774	0.190	0.414	0.886	0.987	0.999	1.000
	GG vs. AA	0.042^a^	2.92 (1.04–8.23)	0.554	0.551	0.786	0.976	0.998	1.000	1.000
	GA/GG vs. AA	0.008^b^	1.91 (1.19–3.08)	0.860	0.129	0.309	0.831	0.980	0.998	1.000
	rs45570941									
	CG/CC vs. GG	0.046^a^	0.60 (0.36–0.99)	0.523	0.292	0.553	0.932	0.993	0.999	1.000
	rs7191779									
	CC vs. GG	0.021^a^	2.06 (1.12–3.82)	0.662	0.291	0.552	0.931	0.993	0.999	1.000
	CC vs. CG/GG	0.013^b^	1.68 (1.12–2.53)	0.825	0.117	0.285	0.814	0.978	0.998	1.000
	rs8044719									
	GT vs. GG	0.035^a^	0.62 (0.40–0.97)	0.566	0.225	0.466	0.906	0.990	0.999	1.000
	GT/TT vs. GG	0.026	0.61 (0.40–0.94)	0.629	0.186	0.407	0.883	0.987	0.999	1.000
	rs8052334									
	TT vs. CC	0.022^a^	2.05 (1.11–3.80)	0.655	0.295	0.556	0.932	0.993	0.999	1.000
	TT vs. TC/CC	0.013^b^	1.68 (1.12–2.54)	0.825	0.120	0.289	0.817	0.978	0.998	1.000
Preeclampsia or preeclampsia superimposed on chronic hypertension (PE/PESCH)	rs10636									
	GC vs. CC	0.003^b^	0.22 (0.08–0.60)	0.949	0.384	0.652	0.954	0.995	1.000	1.000
	CG/GG vs. CC	0.019^a^	0.43 (0.21–0.87)	0.659	0.343	0.611	0.945	0.994	0.999	1.000
	GG vs. CG/CC	0.008^b^	2.03 (1.21–3.41)	0.891	0.154	0.354	0.858	0.984	0.998	1.000
	rs11076161									
	GG vs. GA	0.009^b^	1.95 (1.18–3.22)	0.852	0.151	0.348	0.854	0.983	0.998	1.000
	GG vs. GA/AA	0.005^b^	1.94 (1.22–3.09)	0.909	0.100	0.249	0.785	0.974	0.997	1.000
	rs11644094									
	AG vs. AA	0.050^b^	1.70 (1.00–2.89)	0.662	0.318	0.583	0.939	0.994	0.999	1.000
	AG/GG vs. AA	0.045^b^	1.69 (1.01–2.83)	0.600	0.296	0.557	0.933	0.993	0.999	1.000
	rs8044719									
	GT vs. GG	0.033^a^	0.58 (0.35–0.96)	0.554	0.259	0.512	0.920	0.991	0.999	1.000
	GT/TT vs. GG	0.016^a^	0.55 (0.33–0.89)	0.664	0.180	0.397	0.879	0.987	0.999	1.000
	rs8049883									
	GA vs. AA	0.042^a^	0.49 (0.24–0.98)	0.550	0.407	0.673	0.958	0.996	1.000	1.000
	rs8052334									
	TT vs. TC	0.025^a^	1.62 (1.06–2.48)	0.591	0.175	0.389	0.875	0.986	0.999	1.000
	TT vs. TC/CC	0.045^a^	1.49 (1.01–2.20)	0.520	0.208	0.441	0.897	0.989	0.999	1.000
Hypertensive disorders of pregnancy (HDP)	rs10636									
	GG vs. CC	0.038^a^	0.54 (0.30–0.97)	0.554	0.330	0.597	0.942	0.994	0.999	1.000
	CG/GG vs. CC	0.043^a^	0.55 (0.31–0.98)	0.544	0.338	0.605	0.944	0.994	0.999	1.000
	rs11076161									
	GG vs. AA	0.006^b^	2.02 (1.22–3.36)	0.911	0.133	0.314	0.834	0.981	0.998	1.000
	GG vs. GA/AA	0.000^b^	1.89 (1.35–2.63)	0.993	0.004	**0.013**	0.126	0.592	0.936	0.993
	rs45570941									
	CG vs. GG	0.012^a^	0.61 (0.41–0.90)	0.386	0.104	0.258	0.793	0.975	0.997	1.000
	CG/CC vs. GG	0.014^a^	0.62 (0.43–0.91)	0.306	0.119	0.289	0.817	0.978	0.998	1.000
	rs7191779									
	CC vs. CG	0.018^b^	1.52 (1.08–2.15)	0.466	0.103	0.256	0.791	0.974	0.997	1.000
	CC vs. CG/GG	0.010^b^	1.54 (1.11–2.13)	0.870	0.062	**0.167**	0.687	0.957	0.996	1.000
	rs8044719									
	GT vs. GG	0.001^a^	0.57 (0.40–0.80)	0.606	0.018	**0.052**	0.375	0.858	0.984	0.998
	GT/TT vs. GG	0.028^b^	0.65 (0.44–0.95)	0.484	0.158	0.360	0.861	0.984	0.998	1.000
	rs8049883									
	GA/AA vs. GG	0.017^b^	1.50 (1.08–2.09)	0.858	0.094	0.237	0.774	0.972	0.997	1.000
	rs8052334									
	TT vs. TC	0.017^b^	1.53 (1.08–2.16)	0.477	0.098	0.247	0.783	0.973	0.997	1.000
	TT vs. TC/CC	0.012^b^	1.52 (1.10–2.11)	0.849	0.072	**0.189**	0.719	0.963	0.996	1.000
	rs8052394									
	GA vs. AA	0.037^b^	0.69 (0.49–0.98)	0.701	0.165	0.373	0.867	0.985	0.998	1.000
	GA/GG vs. AA	0.028^b^	0.69 (0.50–0.96)	0.753	0.128	0.306	0.829	0.980	0.998	1.000
	rs9936741									
	CT vs. TT	0.029^a^	0.67 (0.47–0.96)	0.305	0.148	0.343	0.852	0.983	0.998	1.000
	CT/CC vs. TT	0.016^a^	0.65 (0.46–0.92)	0.460	0.098	0.246	0.782	0.973	0.997	1.000

a Unadjusted for age and BMI in a logistic regression model in Table 2 and Table 3.

b Adjusted for age and BMI in a logistic regression model in Table 2 and Table 3.

Figures in bold type indicate statistical significance at the 0.2 level.

### The Linkage Disequilibrium and the Haplotype Analysis

The LD is assessed with the coefficient of linkage disequilibrium (|D’|) and the squared Pearson’s coefficient of correlation (*r* ^2^) (Aumer et al., 2019). The SNPs with |D’| > 0.7 and *r* ^2^ > 1/3 were chosen as tagSNP to conduct haplotype analysis. If *r* ^2^ > 0.8, the two SNPs should be randomly picked up as one tagSNP(El et al., 2021; He et al., 2021; Yoshida et al., 2019). The LD analysis among the 17 designated SNPs in *MT* genes in PCH, GH, PE/PESCH, and HDP and their corresponding controls are shown in Supplementary Figures 1–4, respectively. There were 11 (rs11076161 G > A, rs11640851 C > A, rs1580833 T > G, rs1610216 C > T, rs1827208 C > T, rs7191779 C > G, rs7196890 C > A, rs8044719 G > T, rs8049883 G > A, rs8052394 C > T, and rs9936741 T > C), 10 (rs10636 C > G, rs11076161 G > A, rs11640851 C > A, rs1580833 T > G, rs1610216 C > T, rs28366003 A > G, rs7191779 C > G, rs7196890 C > A, rs8044719 G > T, and rs8049883 G > A), 10 (rs11076161 G > A, rs11640851 C > A, rs1580833 T > G, rs1610216 C > T, rs1827208 C > T, rs7191779 C > G, rs7196890 C > A, rs8044719 G > T, rs8049883 G > A, and rs9936741 T > C), and seven (rs11076161 G > A, rs11640851 C > A, rs1580833 T > G, rs1610216 C > T, rs7191779 C > G, rs7196890 C > A, and rs8049883 G > A) independent SNPs that were identified for haplotype analysis for PCH, GH, PE/PESCH, and HDP, respectively. The global *p*-value of the analysis for each group was lower than 0.0001. It turned out that eight (A-C-G-T-C-G-A-G-C-G-C, OR = 4.559; A-C-T-C-C-C-A-G-C-G-C, OR = 5.777; A-C-T-T-C-G-A-G-C-G-C, OR = 4.590; G-A-T-C-C-G-C-G-G-C-C, OR = 4.065; G-A-T-C-G-C-C-G-G-C-C, OR = 4.652; G-A-T-T-C-C-C-G-G-C-C, OR = 0.404; G-C-T-C-C-C-A-G-G-C-C, OR = 1.901; G-C-T-T-C-C-A-G-G-C-C, OR = 3.810), five (C-G-A-T-C-A-C-C-G-G, OR = 2.032; C-G-A-T-C-G-C-C-G-G, OR = 2.077; G-A-C-T-C-A-C-C-T-G, OR = 0.564; G-G-A-G-C-A-C-C-G-G, OR = 5.466; G-G-A-T-T-A-G-C-G-G, OR = 0.284), five (A-C-G-T-C-G-A-G-C-C, OR = 2.399; A-C-T-C-C-C-C-T-G-G, OR = 0.259; G-A-T-C-C-C-C-G-G-C, OR = 1.572; G-A-T-C-G-C-C-G-G-C, OR = 0.001; G-C-T-C-G-C-A-G-G-C, OR = 2.512), and five (A-C-T-C-C-C-G, OR = 0.634; G-A-G-C-C-C-G, OR = 4.047; G-A-T-T-G-C-G, OR = 0.499; G-C-G-T-C-A-G, OR = 7.299; G-C-T-C-C-A-G, OR = 1.434) haplotypes were significantly associated with PCH, GH, PE/PESCH, and HDP, respectively (Table 5).

**TABLE 5:** Haplotype analysis of designated variations in *MT* genes among cases and controls.

Group		Haplotype^b^	Case^a^ (frequencies)	Control^a^ (frequencies)	χ^2^	*p*-value	Odds ratio [95% CI]
Pregnancy with chronic hypertension (PCH)	1	A-C-G-T-C-G-A-G-C-G-C	9.52 (0.074)	15.51 (0.017)	15.245	**0.000**	4.559 [1.988–10.458]
	2	A-C-T-C-C-C-A-G-C-G-C	2.14 (0.017)	2.62 (0.003)	4.576	**0.032**	5.777 [0.939–35.539]
	3	A-C-T-T-C-G-A-G-C-G-C	5.22 (0.041)	8.21 (0.009)	8.622	**0.003**	4.590 [1.507–13.978]
	4	G-A-T-C-C-G-C-G-G-C-C	5.40 (0.042)	9.59 (0.010)	7.670	**0.006**	4.065 [1.392–11.870]
	5	G-A-T-C-G-C-C-G-G-C-C	20.30 (0.159)	34.95 (0.037)	31.274	**0.000**	4.652 [2.594–8.342]
	6	G-A-T-T-C-C-C-G-G-C-C	8.41 (0.066)	132.06 (0.141)	6.406	**0.011**	0.404 [0.196–0.833]
	7	G-C-T-C-C-C-A-G-G-C-C	14.45 (0.113)	56.16 (0.060)	4.363	**0.037**	1.901 [1.031–3.504]
	8	G-C-T-T-C-C-A-G-G-C-C	6.93 (0.054)	13.25 (0.014)	8.950	**0.003**	3.810 [1.490–9.744]
Gestational hypertension GH)	1	C-G-A-T-C-A-C-C-G-G	29.97 (0.092)	43.36 (0.046)	8.476	**0.004**	2.032 [1.250–3.303]
	2	C-G-A-T-C-G-C-C-G-G	38.04 (0.117)	54.73 (0.059)	11.133	**0.001**	2.077 [1.342–3.214]
	3	G-A-C-T-C-A-C-C-T-G	18.01 (0.056)	85.27 (0.091)	4.669	**0.031**	0.564 [0.333–0.954]
	4	G-G-A-G-C-A-C-C-G-G	4.04 (0.012)	2.09 (0.002)	4.969	**0.026**	5.466 [1.023–29.202]
	5	G-G-A-T-T-A-G-C-G-G	5.84 (0.018)	54.78 (0.059)	9.246	**0.002**	0.284 [0.120–0.673]
Preeclampsia or preeclampsia superimposed on chronic hypertension (PE/PESCH)	1	A-C-G-T-C-G-A-G-C-C	11.56 (0.046)	18.15 (0.020)	5.510	**0.019**	2.399 [1.131–5.091]
	2	A-C-T-C-C-C-C-T-G-G	5.19 (0.021)	68.84 (0.074)	9.863	**0.002**	0.259 [0.105–0.640]
	3	G-A-T-C-C-C-C-G-G-C	41.65 (0.167)	103.73 (0.112)	5.135	**0.023**	1.572 [1.060–2.330]
	4	G-A-T-C-G-C-C-G-G-C	0.01 (0.000)	27.63 (0.030)	6.796	**0.009**	0.001 [0.000–0.016]
	5	G-C-T-C-G-C-A-G-G-C	22.94 (0.092)	35.52 (0.038)	11.571	**0.001**	2.512 [1.454–4.340]
Hypertensive disorders of pregnancy (HDP)	1	A-C-T-C-C-C-G	49.47 (0.070)	98.13 (0.104)	6.342	**0.012**	0.634 [0.443–0.906]
	2	G-A-G-C-C-C-G	11.22 (0.016)	3.68 (0.004)	6.285	**0.012**	4.047 [1.241–13.192]
	3	G-A-T-T-G-C-G	21.07 (0.030)	53.66 (0.057)	7.280	**0.007**	0.499 [0.298–0.834]
	4	G-C-G-T-C-A-G	10.50 (0.015)	1.91 (0.002)	8.695	**0.003**	7.299 [1.556–34.230]
	5	G-C-T-C-C-A-G	108.76 (0.153)	104.44 (0.111)	5.994	**0.014**	1.434 [1.073–1.914]

PCH is associated with haplotypes derived among rs11076161 G > A, rs11640851 C > A, rs1580833 T > G, rs1610216 C > T, rs1827208 C > T, rs7191779 C > G, rs7196890 C > A, rs8044719 G > T, rs8049883 G > A, rs8052394 C > T, and rs9936741 T > C.

GH is associated with haplotypes derived among rs10636 C > G, rs11076161 G > A, rs11640851 C > A, rs1580833 T > G, rs1610216 C > T, rs28366003 A > G, rs7191779 C > G, rs7196890 C > A, rs8044719 G > T, and rs8049883 G > A.

PE/PESCH is associated with haplotypes derived among rs11076161 G > A, rs11640851 C > A, rs1580833 T > G, rs1610216 C > T, rs1827208 C > T, rs7191779 C > G, rs7196890 C > A, rs8044719 G > T, rs8049883 G > A, and rs9936741 T > C.

HDP is associated with haplotypes derived among rs11076161 G > A, rs11640851 C > A, rs1580833 T > G, rs1610216 C > T, rs7191779 C > G, rs7196890 C > A, and rs8049883 G > A.

a The numbers are the frequencies of the haplotypes in 66 PCH, 172 GH, 133 PE, and 371 HDP cases and their corresponding controls.

b Haplotypes were eliminated if the estimated haplotype probability (in parentheses) is less than 0.01.

Figures in bold type indicate statistical significance at the 0.05 level.

## Discussion

In the present study, rs10636 (*MT2A*) was identified to be significantly associated with GH risk. Other four SNPs, that is, rs11076161 (*MT1A*), rs7191779 (*MT1B*), rs8044719 (*MT1DP*), and rs8052334 (*MT1B*) in *MT* genes were identified to be significantly associated with HDP susceptibility. A joint effect of 11, 10, 10, and 7 SNPs in *MT* genes including *MT1A*, *MT2A*, *MT1M*, *MT1B*, and *MT1DP* on HDP susceptibility was identified in the haplotype analysis, showing that eight, five, five, and five haplotypes of *MT* alleles were significantly associated with the risks of PCH, GH, PESCH, and HDP, respectively.

Of the SNPs in *MT* genes associated with HDP susceptibility, both rs11076161 in *MT1A* and rs8052334 in *MT1B* are intron-located SNPs, which may modulate gene expression in a cumulative manner (Goebels et al., 2013). In addition, the SNP Function Prediction (FuncPred) showed that rs11076161 was related to the transcription factor binding site (TFBS). The genetic variations at the site may influence gene expression by altering the interaction between transcription factors and TFBS (Lai et al., 2019). This might affect the function of MT1A as an antioxidant and ultimately disturb the cellular redox homeostasis, and that may be one of the potential mechanisms by which the genetic variations in *MT* genes modify the individual susceptibility to HDP. Our study revealed that the HDP risk of rs11076161 GG variants was 1.89 times as high as that of the GA/AA alleles, while the HDP risk of rs8052334 TT variants was 1.52 times as high as that of TC/CC variants. It has been shown that rs11076161 is significantly associated with the oxidation-related diabetic neuropathy in patients with type 2 diabetes and the body’s load of cadmium (Lei et al., 2012) and that rs8052334 is associated to a decreased utilization of fatty acid (hyperlipidemia) in type 2 diabetes (Yang et al., 2008). The *MT-1* A-G-T haplotypes (*MT1A* rs8052394 A allele, *MT1B* rs964372 G allele, and *MT1B* rs8052334 T allele) are related to an increased susceptibility of hepatocellular carcinoma (HCC), especially in those who smoke, presenting a notable combined effect of cigarette smoking and *MT-1* haplotypes on HCC development (Wong et al., 2013).

The SNP rs10636 is located at the 3′ UTR region in the *MT2A* gene. Importantly, the 3′ UTR regions are often highly conserved and play an important role in modulating protein abundance through the regulation of translation or stability of mRNAs (Mayr, 2019). FuncPred analysis showed that rs10636 is related to TFBS as functioning with splicing and miRNA regulations. In our study, rs10636 G variants were identified to be associated with a decreased risk of GH. This result is consistent with the work of Liu et al. (2017) in which the genotype GG of rs10636 has a protective role in GH (Liu et al., 2017). The genetic combination of rs10636 GC-rs28366003 AA in *MT2A* presented less susceptibility to lead (Pb)-induced lipid oxidation than other genotypes (Yang et al., 2017).

*MT* expression is mainly regulated at the transcriptional level (Raudenska et al., 2014). In addition to the basal regulatory elements such as the TATA-box, GC-box, and basal level enhancer (BLE) sequences in the upstream of *MT* genes, there exist several inducible elements including metal response elements (MREs), antioxidant responsive elements (AREs), and glucocorticoid responsive elements (GRE) for regulating *MT* expression (Dalton et al., 1994; Raudenska et al., 2014). The SNP rs7191779 is located at 2 kb upstream of the *MT1B* gene and is related to the TFBS in FuncPred analyses. Our data showed that people with the CC genotype of rs7191779 demonstrated a 1.43-fold risk of those with GG/CG to develop HDP, suggesting that genetic variations may modify the susceptibility of HDP probably by regulating *MT* expression during pregnancy, which is more easily subjected to oxidative stress. It has been shown that some other oxidation-related conditions are also associated with the genetic variations. For example, the rs7191779 CC genotypes are presented as the risk factors of age-related macular degeneration (Garcia et al., 2017) and type 2 diabetes mellitus (Yang et al., 2008), whereas the rs7191779 GC genotypes experience significant protection against oral squamous cell carcinoma development (Zavras et al., 2011).

One of the most intriguing characteristics of *MTs* is the existence of 7 nonfunctional alleles in addition to 12 functional isoforms (Raudenska et al., 2014; McNeill et al., 2019). The *MT1D* has been predicted to be a non-active form (Gai et al., 2020). However, there exists an *MT1D* pseudogene (*MT1DP*) that is identified as a long noncoding RNA (lncRNA), which functions to aggravate oxidative stress by repressing antioxidation (Gai et al., 2020) and is known as an oncogene (Liao et al., 2021). *MT1DP* is proposed to be a result of gene duplicates to preserve the parental function and acts as backup compensation copies to buffer against the loss of a functionally related gene (Moleirinho et al., 2011). Liao et al. (2021)found that the combination of lncRNA *MT1DP* and miR-365 damaged the cell mitochondrial membrane to reduce mitochondrial function and the ability of ROS elimination and increased cell apoptosis. In our study, the SNP rs8044719 that is located at 500 bp downstream of *MT1DP* was associated with a decreased HDP risk. The carriers of the GT genotypes of rs8044719 were less likely to develop HDP than those carrying GG (OR = 0.57 for GT vs. GG). It is hence speculated that the GT genotypes of rs8044719 may attenuate the effect of *MT1DP* on repressing antioxidation to reduce the risk of HDP development.

The association of variations in *MT* genes with HDP was assessed by different approaches in this work. In the logistic regression model, two, seven, six, and seven SNPs were presented to be associated with the susceptibility of PCH, GH, PE/PESCH, and HDP, respectively. After tested by FPRP analysis, rs11076161 in *MT1A* and rs8044719 in *MT1DP* were related to PCH, whereas only rs10636 in *MT2A* was associated with GH. The association of *MT* variations with PE/PESCH disappeared. Four SNPs, that is, rs11076161 (*MT1A*), rs7191779 (*MT1B*), rs8044719 (*MT1DP*), and rs8052334 (*MT1B*) were associated with HDP. FPRP confirmed the noteworthy findings in the current study. Even in Bonferroni’s, test that is a very conservative correction procedure (White et al., 2019; Zill et al., 2004), rs8044719 and rs11076161 were still recognized to be associated with HDP. The statistical power was generally set as 80% for determining the sample size that may yield the acceptable probability estimates (Hong and Park, 2012). In this study, the *post hoc* power analysis showed that the power of rs10636 (GG vs. GC; GG vs. CG/CC), rs11076161 (GG vs. GA/AA), rs7191779 (CC vs. CG/GG), or rs8052334 (TT vs. TC/CC) was greater than 0.8. Although the power of rs8044719 (GT vs. GG) was 0.612, the association of the SNP with HDP risk was further tested with FPRP and maintained statistically significance. On the other hand, the powers of rs11076161 (GG vs. GA/AA) and rs8044719 (GT/TT vs. GG) related to PCH were low (0.180 and 0.052, respectively) as the probability of detecting the association might not be acceptable.

Haplotype analysis was used to identify the joint effects among studied SNPs. We found that the frequencies of seven haplotypes of *MT* alleles were higher and one haplotype was lower in PCH and the frequencies of three haplotypes were higher and two haplotypes were lower in GH, PESCH, and HDP than that in their corresponding controls. This evidence reveals not only the single-locus effect but a joint effect of SNPs in different *MT* genes on HDP susceptibility, yet the mechanisms remain to be further elucidated. It has been shown that the differential expression levels of MT isoforms present in different diseases and tumor types (Aquime et al., 2020; Hung et al., 2019; Saiegh et al., 2019; Sampaio et al., 2019), suggesting that MT isoforms may play distinct roles in pathophysiological processes under extreme conditions. *MTs* are highly evolutionarily conserved but non-essential; the antioxidative action of MTs is far stronger than that of superoxide dismutase (SOD) and GSH (Ma'Rifah et al., 2019; Thornalley and Vasak, 1985), and the latter is required for the recycling of oxidative and reduced forms of MTs (Kang, 2006). It, thus, appeared that MTs act as a backup of antioxidative systems, providing an amplification of the antioxidant activity in working with other antioxidants such as GSH and Nrf2 (Gu et al., 2017). The variation rs8044719 located at an *MT* pseudogene (*MT1DP*) was also considered to be a significant genetic factor affecting HDP susceptibility. This finding is reminiscent of the implication that the coexistence of the original and pseudogene genes may be related to the reconstruction and evolutionary history of the *MT* gene family (Moleirinho et al., 2011).

HDP has a common feature that is elevated blood pressure. Furthermore, PE has proteinuria (Phoswa, 2019). This implies that there exist different pathogenic contexts of PE from PCH or GH. Our data supported the notion by presenting that seven independent SNPs (rs11076161 G > A, rs11640851 C > A, rs1580833 T > G, rs1610216 C > T, rs7191779 C > G, rs7196890 C > A, and rs8049883 G > A) were related to all three subtypes of HDP in haplotype analysis, but no single SNP in *MT* genes is simultaneously associated with all subtypes. PE has been proposed to reduce uteroplacental perfusion as a result of abnormal cytotrophoblast invasion of spiral arterioles (Phoswa and Khaliq, 2021) and is closely related to oxidative stress in the placenta (Vitoratos et al., 2012), indicating more complicated etiological processes than that in other subtypes of HDP. In a study on the effect of SNPs in the renin–angiotensin–aldosterone system (RAAS) genes on HDP risks, it was shown that rs3789678 in the angiotensinogen gene was significantly associated with GH, whereas rs275645 in the angiotensin II receptor type 1 gene was significantly associated with PE (Li et al., 2016).

There are some advantages in our study. The associations between *MT* polymorphisms and HDP susceptibility were estimated with diverse methods, including logistic regression analysis and haplotype analysis, for accessing the possible effect of a single SNP or joint effects of studied multiple genetic variations on HDP risks. Moreover, the associations were further confirmed by FPRP analyses. However, this study also has several limitations. First, this study was a hospital-based case-control study design, which may be affected by selection bias. Second, the selected SNPs do not include all functional SNPs in *MT* genes, which results in missing some meaningful information for functional *MTs*. Third, the study lacked the ability to assess gene–environment interactions. A nested case-control study based on the prospective cohort with a larger sample size should be conducted to further elucidate the issues.

## Conclusion

The genetic variation rs10636 G in *MT2A* was related to a decreased risk of gestational hypertension. Other four SNPs (rs11076161 in *MT1A*, rs7191779 in *MT1B*, rs8044719 in *MT1DP*, and rs8052334 in *MT1B*) in *MT* genes are individually associated with HDP susceptibility. The frequencies of seven haplotypes of *MT* alleles were higher and those of one haplotype were lower in PCH, and the frequencies of three haplotypes were higher and those of two haplotypes were lower in GH, PESCH, and HDP than those in their corresponding controls. These variations and their haplotypes may be used as early warning genetic markers to predict HDP for women of childbearing age at high risk.

## Data Availability

The original contributions presented in the study are publicly available. This data can be found on Dryad with the doi:10.5061/dryad.1c59zw3xz
