# Supplementary figures and images for: Genetic Variations in Metallothionein Genes and Susceptibility to Hypertensive Disorders of Pregnancy: A Case-Control Study
Source: Front Genet. 2022 Jun 6;13:830446. doi: 10.3389/fgene.2022.830446 (PMC9208279; doi:10.3389/fgene.2022.830446)

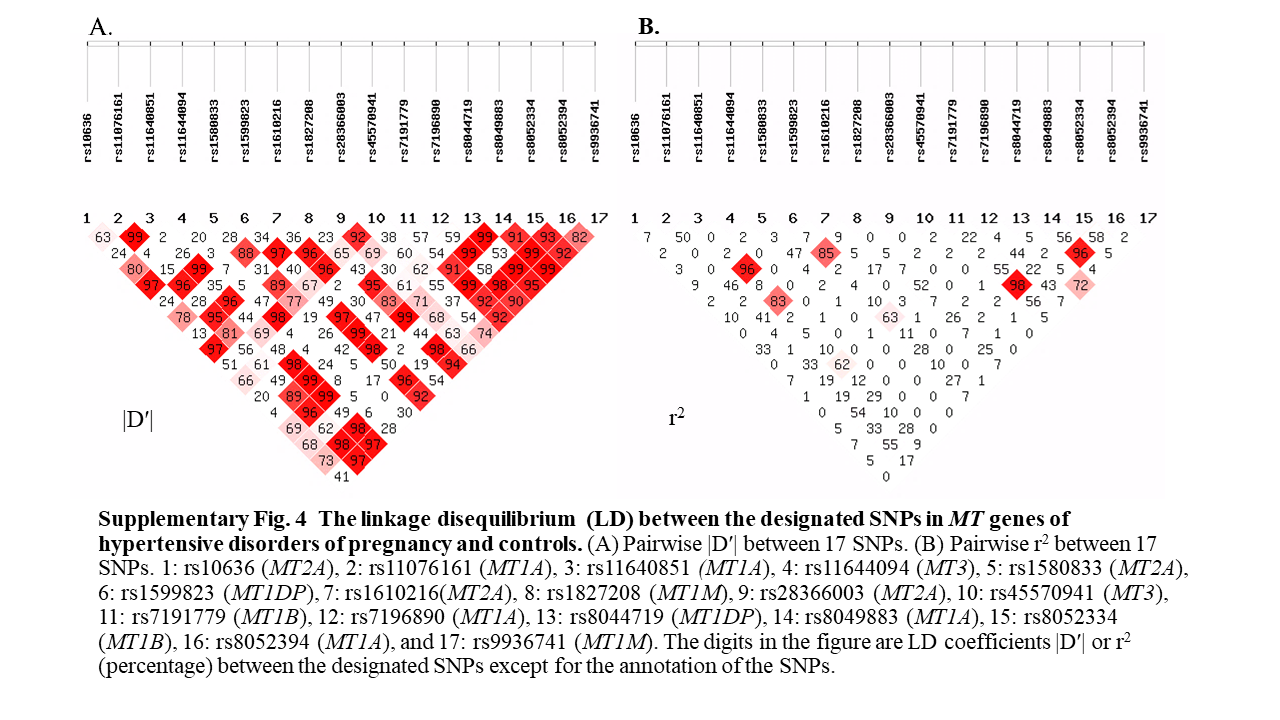

Supplement: Supplementary file 1 [file Image4.PNG]

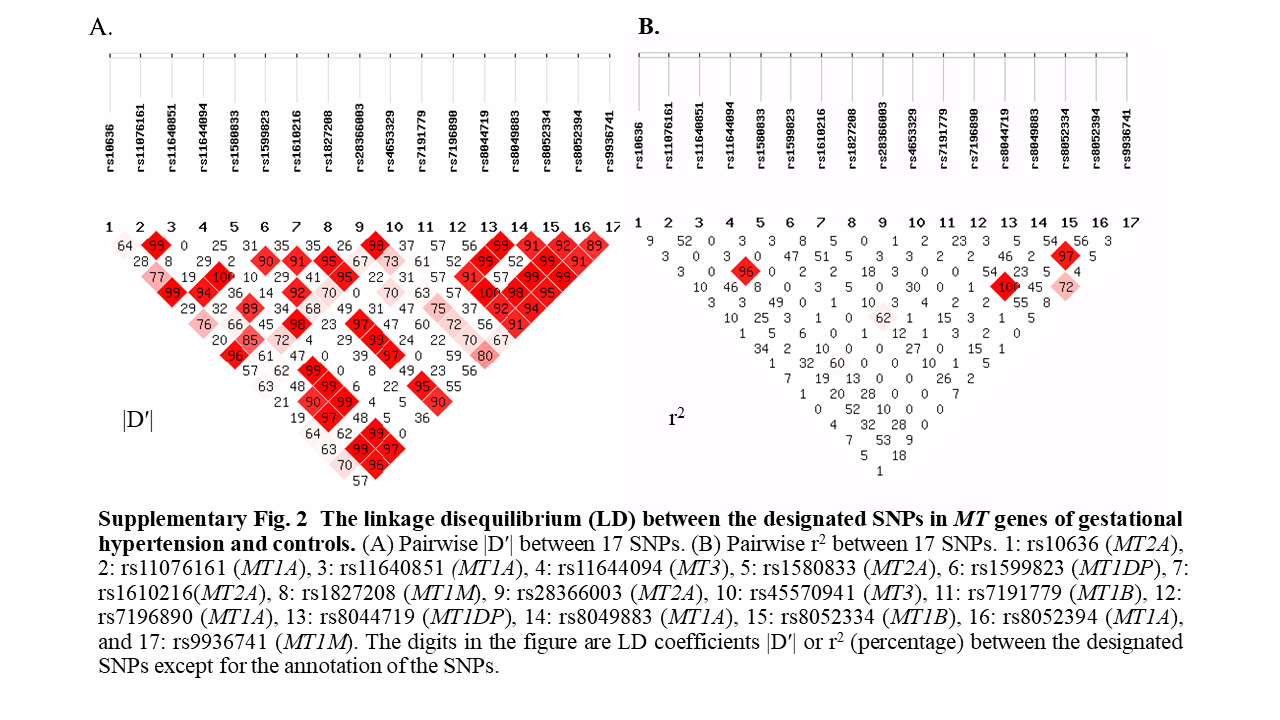

Supplement: Supplementary file 2 [file Image2.PNG]

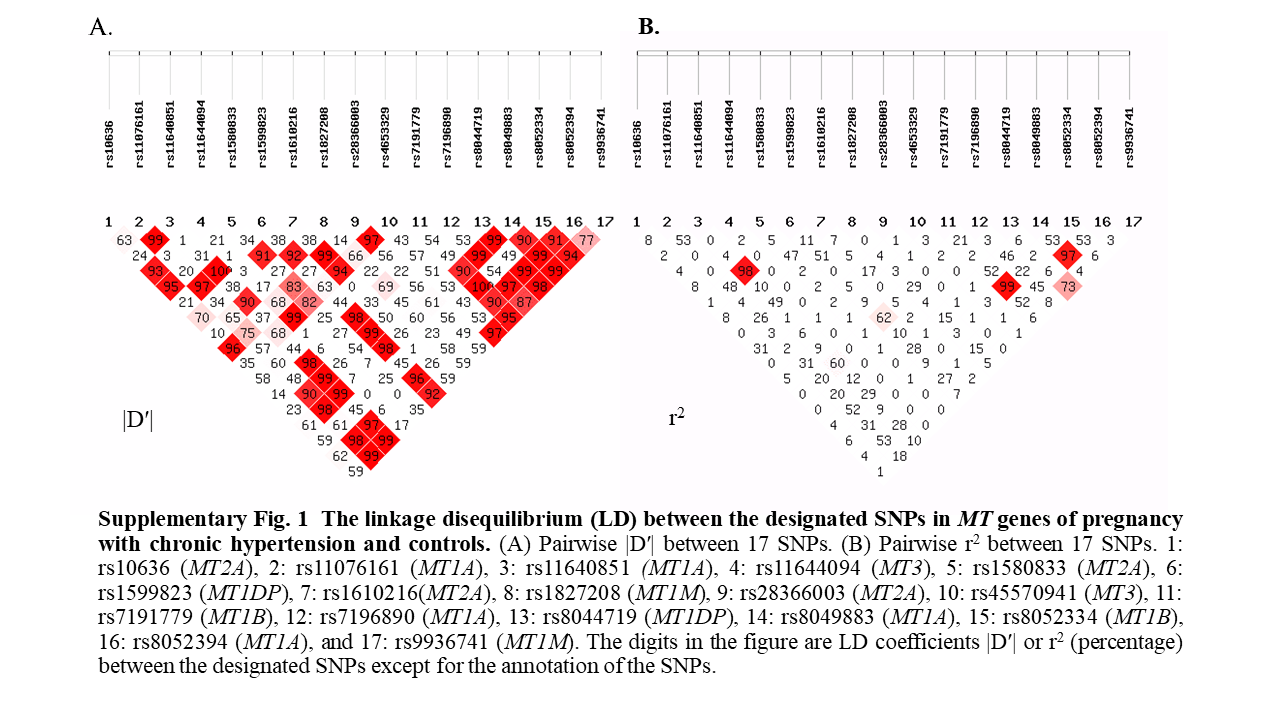

Supplement: Supplementary file 3 [file Image1.PNG]

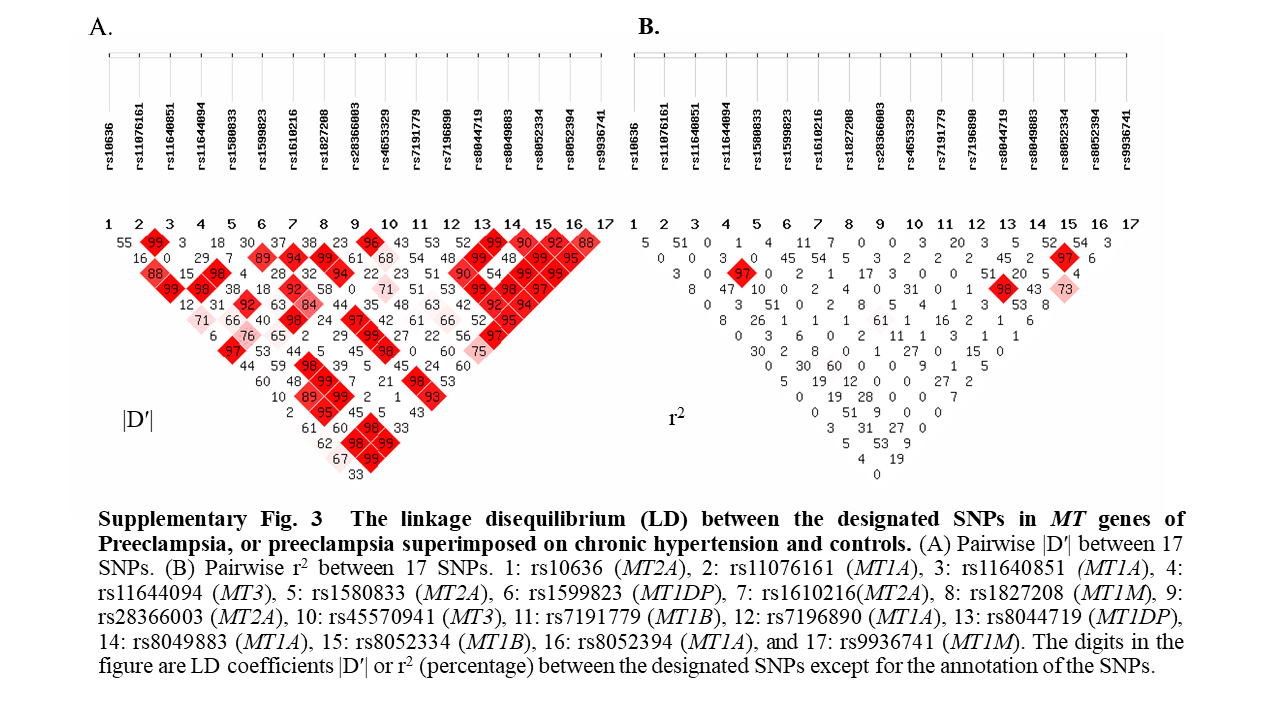

Supplement: Supplementary file 4 [file Image3.PNG]
