# Supplementary material for: Genetic Variations in Metallothionein Genes and Susceptibility to Hypertensive Disorders of Pregnancy: A Case-Control Study
Source: Front Genet. 2022 Jun 6;13:830446. doi: 10.3389/fgene.2022.830446 (PMC9208279; doi:10.3389/fgene.2022.830446)
Supplement: Supplementary file 5 [file DataSheet1.docx]

**Supplementary table 1 The information of designated SNPs in *MT* genes**

| Gene | SNP | Flanking sequence | Ancestral allele | Functional region |
| --- | --- | --- | --- | --- |
| *MT2A* | rs10636 | TGACC[**G/C**]TGACC | C | 3' UTR |
| *MT1A* | rs11076161 | ATAAA[**A/G**]TTCAC | A | intron |
| *MT1A* | rs11640851 | ATGCA[**C/A**]CTCCT | C | missense |
| *MT3* | rs11644094 | CTCCA[**A/G**]TGGCA | A | intron |
| *MT2A* | rs1580833 | CACTT[**G/T**]CTGGC | T | NA |
| *MT1DP* | rs1599823 | AACTC[**T/C**]TTGAC | G | 2kb upstream |
| *MT2A* | rs1610216 | CCCCG[**A/G**]GGCGC | C | 2kb upstream |
| *MT1M* | rs1827208 | TCCCG[**C/T**]TCCAC | C | intron |
| *MT2A* | rs28366003 | GCTGC[**A/G**]CTCCA | A | 2kb upstream |
| *MT3* | rs45570941 | TTACG[**G/C**]ATCCT | G | intron |
| *MT1B* | rs7191779 | CCAAG[**C/G**]TGTGT | G | 2kb upstream |
| *MT1A* | rs7196890 | TGTGC[**C/A**]TTGGA | C | 2kb upstream |
| *MT1DP* | rs8044719 | CTCTG[**T/G**]TTTTT | G | 500b downstream |
| *MT1A* | rs8049883 | GTTTC[**G/A**]TTCTT | A | intron |
| *MT1B* | rs8052334 | CAGGC[**T/C**]TGCTG | C | intron |
| *MT1A* | rs8052394 | CTGCA[**A/G**]AGGGG | A | missense |
| *MT1M* | rs9936741 | GTTAA[**T/C**]AGAAC | T | 3' UTR |

**Supplementary table 2 The genotype frequencies of the designated SNPs in studied populations**

|  | Gene | SNP genotype | Call rate (%) | control (n) | HDP (n) | *P*^a^ | PCH (n) | *P*^b^ | GH (n) | *P*^c^ | PE/PESCH (n) | *P*^d^ |
| --- | --- | --- | --- | --- | --- | --- | --- | --- | --- | --- | --- | --- |
| 1 | *MT2A* | rs10636 | 98.2 |  |  |  |  |  |  |  |  |  |
|  |  | CC |  | 22 | 29 | 0.110 | 7 | 0.079 | 9 | **0.000** | 13 | **0.000** |
|  |  | GC |  | 169 | 129 |  | 18 |  | 94 |  | 17 |  |
|  |  | GG |  | 284 | 202 |  | 40 |  | 65 |  | 97 |  |
|  |  | GC/GG^e^ |  | 453 | 331 | **0.041** | 58 | 0.069 | 159 | 0.706 | 114 | **0.017** |
|  |  | GG/CC |  | 191 | 158 |  | 25 |  | 103 |  | 30 |  |
|  |  | GG^f^ |  | 284 | 202 | 0.286 | 40 | 0.787 | 65 | **0.000** | 97 | **0.001** |
| 2 | *MT1A* | rs11076161 | 99.4 |  |  |  |  |  |  |  |  |  |
|  |  | AA |  | 75 | 48 | **0.001** | 14 | **0.035** | 16 | **0.014** | 18 | **0.003** |
|  |  | GA |  | 243 | 150 |  | 22 |  | 79 |  | 49 |  |
|  |  | GG |  | 159 | 170 |  | 29 |  | 76 |  | 65 |  |
|  |  | GA/GG^e^ |  | 402 | 320 | 0.273 | 51 | 0.235 | 155 | **0.040** | 114 | 0.555 |
|  |  | GA/AA |  | 318 | 198 |  | 36 |  | 95 |  | 67 |  |
|  |  | GG^f^ |  | 159 | 170 | **0.000** | 29 | 0.073 | 76 | **0.010** | 65 | **0.001** |
| 3 | *MT1A* | rs11640851 | 99.5 |  |  |  |  |  |  |  |  |  |
|  |  | CC |  | 148 | 106 | 0.308 | 24 | 0.431 | 41 | 0.153 | 41 | 0.555 |
|  |  | CA |  | 243 | 181 |  | 28 |  | 91 |  | 62 |  |
|  |  | AA |  | 86 | 82 |  | 14 |  | 39 |  | 29 |  |
|  |  | CA/AA^e^ |  | 329 | 263 | 0.469 | 42 | 0.382 | 130 | 0.082 | 91 | 0.994 |
|  |  | CA/CC |  | 391 | 287 |  | 52 |  | 132 |  | 103 |  |
|  |  | AA^f^ |  | 86 | 82 | 0.130 | 14 | 0.532 | 39 | 0.174 | 29 | 0.306 |
| 4 | *MT3* | rs11644094 | 99.2 |  |  |  |  |  |  |  |  |  |
|  |  | AA |  | 357 | 257 | 0.251 | 46 | 0.747 | 126 | 0.877 | 85 | 0.068 |
|  |  | AG |  | 110 | 100 |  | 16 |  | 42 |  | 42 |  |
|  |  | GG |  | 9 | 10 |  | 2 |  | 4 |  | 4 |  |
|  |  | AG/GG^e^ |  | 119 | 110 | 0.108 | 18 | 0.590 | 46 | 0.653 | 46 | **0.021** |
|  |  | AG/AA |  | 467 | 357 |  | 62 |  | 168 |  | 127 |  |
|  |  | GG^f^ |  | 9 | 10 | 0.419 | 2 | 0.628 | 4 | 0.753 | 4 | 0.492 |
| 5 | *MT2A* | rs1580833 | 99.2 |  |  |  |  |  |  |  |  |  |
|  |  | TT |  | 283 | 196 | 0.219 | 33 | 0.343 | 95 | 0.319 | 68 | 0.222 |
|  |  | GT |  | 162 | 145 |  | 27 |  | 65 |  | 53 |  |
|  |  | GG |  | 32 | 25 |  | 6 |  | 7 |  | 12 |  |
|  |  | GT/GG^e^ |  | 194 | 170 | 0.093 | 33 | 0.150 | 72 | 0.591 | 65 | 0.091 |
|  |  | TT/GT |  | 445 | 341 |  | 60 |  | 160 |  | 121 |  |
|  |  | GG^f^ |  | 32 | 25 | 0.944 | 6 | 0.443 | 7 | 0.241 | 12 | 0.362 |
| 6 | *MT1DP* | rs1599823 | 69.8 |  |  |  |  |  |  |  |  |  |
|  |  | GG |  | 41 | 83 | 0.124 | 13 | 0.551 | 42 | **0.044** | 28 | 0.386 |
|  |  | AG |  | 122 | 169 |  | 27 |  | 85 |  | 57 |  |
|  |  | AA |  | 79 | 99 |  | 23 |  | 39 |  | 37 |  |
|  |  | AG/AA^e^ |  | 201 | 268 | **0.048** | 50 | 0.494 | 124 | **0.039** | 94 | 0.167 |
|  |  | AG/GG |  | 163 | 252 |  | 40 |  | 127 |  | 85 |  |
|  |  | AA^f^ |  | 79 | 99 | 0.246 | 23 | 0.563 | 39 | **0.045** | 37 | 0.654 |
| 7 | *MT2A* | rs1610216 | 98.8 |  |  |  |  |  |  |  |  |  |
|  |  | CC |  | 201 | 164 | 0.580 | 28 | 0.981 | 84 | 0.137 | 52 | 0.907 |
|  |  | CT |  | 206 | 153 |  | 28 |  | 67 |  | 58 |  |
|  |  | TT |  | 70 | 46 |  | 9 |  | 17 |  | 20 |  |
|  |  | CT/TT^e^ |  | 276 | 199 | 0.378 | 37 | 0.886 | 84 | 0.078 | 78 | 0.661 |
|  |  | CT/CC |  | 407 | 317 |  | 56 |  | 151 |  | 110 |  |
|  |  | TT^f^ |  | 70 | 46 | 0.405 | 9 | 0.859 | 17 | 0.137 | 20 | 0.840 |
| 8 | *MT1M* | rs1827208 | 98.7 |  |  |  |  |  |  |  |  |  |
|  |  | CC |  | 158 | 116 | 0.675 | 26 | 0.568 | 49 | 0.510 | 41 | 0.588 |
|  |  | CT |  | 236 | 179 |  | 29 |  | 88 |  | 62 |  |
|  |  | TT |  | 80 | 70 |  | 10 |  | 33 |  | 27 |  |
|  |  | CT/TT^e^ |  | 316 | 249 | 0.635 | 39 | 0.288 | 121 | 0.280 | 89 | 0.700 |
|  |  | CT/CC |  | 394 | 295 |  | 55 |  | 137 |  | 103 |  |
|  |  | TT^f^ |  | 80 | 70 | 0.389 | 10 | 0.762 | 33 | 0.456 | 27 | 0.303 |
| 9 | *MT2A* | rs28366003 | 99.8 |  |  |  |  |  |  |  |  |  |
|  |  | AA |  | 401 | 291 | 0.188 | 55 | 0.732 | 120 | **0.000** | 116 | 0.201 |
|  |  | GA |  | 70 | 69 |  | 9 |  | 45 |  | 15 |  |
|  |  | GG |  | 8 | 9 |  | 2 |  | 7 |  | 0 |  |
|  |  | GA/GG^e^ |  | 78 | 78 | 0.070 | 11 | 0.937 | 52 | **0.000** | 15 | 0.173 |
|  |  | GA/AA |  | 471 | 360 |  | 64 |  | 165 |  | 131 |  |
|  |  | GG^f^ |  | 8 | 9 | 0.428 | 2 | 0.346 | 7 | 0.081 | 0 | 0.212 |
| 10 | *MT3* | rs45570941 | 99.3 |  |  |  |  |  |  |  |  |  |
|  |  | GG |  | 382 | 319 | **0.041** | 57 | 0.325 | 149 | 0.120 | 113 | 0.214 |
|  |  | CG |  | 87 | 44 |  | 7 |  | 21 |  | 16 |  |
|  |  | CC |  | 7 | 5 |  | 1 |  | 1 |  | 3 |  |
|  |  | CG/CC^e^ |  | 94 | 49 | **0.013** | 8 | 0.150 | 22 | **0.044** | 19 | 0.162 |
|  |  | CG/GG |  | 469 | 363 |  | 64 |  | 170 |  | 129 |  |
|  |  | CC^f^ |  | 7 | 5 | 0.892 | 1 | 0.966 | 1 | 0.688 | 3 | 0.459 |
| 11 | *MT1B* | rs7191779 | 99.1 |  |  |  |  |  |  |  |  |  |
|  |  | GG |  | 65 | 42 | **0.041** | 8 | 0.870 | 15 | **0.011** | 19 | 0.138 |
|  |  | CG |  | 221 | 147 |  | 32 |  | 66 |  | 49 |  |
|  |  | CC |  | 189 | 178 |  | 24 |  | 90 |  | 64 |  |
|  |  | CG/CC^e^ |  | 410 | 325 | 0.333 | 56 | 0.795 | 156 | 0.094 | 113 | 0.835 |
|  |  | CG/GG |  | 286 | 189 |  | 40 |  | 81 |  | 68 |  |
|  |  | CC^f^ |  | 189 | 178 | **0.011** | 24 | 0.725 | 90 | **0.004** | 64 | 0.073 |
| 12 | *MT1A* | rs7196890 | 99.5 |  |  |  |  |  |  |  |  |  |
|  |  | CC |  | 152 | 117 | 0.974 | 17 | 0.419 | 60 | 0.561 | 40 | 0.816 |
|  |  | CA |  | 233 | 176 |  | 32 |  | 82 |  | 62 |  |
|  |  | AA |  | 94 | 74 |  | 17 |  | 28 |  | 29 |  |
|  |  | CA/AA^e^ |  | 327 | 250 | 0.964 | 49 | 0.325 | 110 | 0.395 | 91 | 0.794 |
|  |  | CA/CC |  | 385 | 293 |  | 49 |  | 142 |  | 102 |  |
|  |  | AA^f^ |  | 94 | 74 | 0.846 | 17 | 0.246 | 28 | 0.366 | 29 | 0.525 |
| 13 | *MT1DP* | rs8044719 | 99.5 |  |  |  |  |  |  |  |  |  |
|  |  | GG |  | 344 | 304 | **0.002** | 56 | 0.054 | 139 | 0.081 | 109 | **0.040** |
|  |  | GT |  | 120 | 60 |  | 8 |  | 30 |  | 22 |  |
|  |  | TT |  | 13 | 5 |  | 1 |  | 3 |  | 1 |  |
|  |  | GT/TT^e^ |  | 133 | 65 | **0.000** | 9 | **0.016** | 33 | **0.025** | 23 | **0.015** |
|  |  | GT/GG |  | 464 | 364 |  | 64 |  | 169 |  | 131 |  |
|  |  | TT^f^ |  | 13 | 5 | 0.171 | 1 | 1.000 | 3 | 0.579 | 1 | 0.322 |
| 14 | *MT1A* | rs8049883 | 99.4 |  |  |  |  |  |  |  |  |  |
|  |  | AA |  | 37 | 33 | 0.126 | 8 | 0.462 | 10 | 0.243 | 15 | **0.045** |
|  |  | GA |  | 183 | 117 |  | 24 |  | 57 |  | 36 |  |
|  |  | GG |  | 256 | 219 |  | 33 |  | 105 |  | 81 |  |
|  |  | GA/GG^e^ |  | 439 | 336 | 0.541 | 57 | 0.214 | 162 | 0.396 | 117 | 0.192 |
|  |  | GA/AA |  | 220 | 150 |  | 32 |  | 67 |  | 51 |  |
|  |  | GG^f^ |  | 256 | 219 | 0.106 | 33 | 0.648 | 105 | 0.100 | 81 | 0.121 |
| 15 | *MT1B* | rs8052334 | 98.9 |  |  |  |  |  |  |  |  |  |
|  |  | CC |  | 65 | 42 | **0.035** | 8 | 0.787 | 15 | **0.011** | 19 | 0.077 |
|  |  | TC |  | 224 | 146 |  | 33 |  | 66 |  | 47 |  |
|  |  | TT |  | 188 | 176 |  | 23 |  | 89 |  | 64 |  |
|  |  | TC/TT^e^ |  | 412 | 322 | 0.368 | 56 | 0.804 | 155 | 0.102 | 111 | 0.772 |
|  |  | TC/CC |  | 289 | 188 |  | 41 |  | 81 |  | 66 |  |
|  |  | TT^f^ |  | 188 | 176 | **0.010** | 23 | 0.592 | 89 | **0.003** | 64 | **0.044** |
| 16 | *MT1A* | rs8052394 | 99.6 |  |  |  |  |  |  |  |  |  |
|  |  | AA |  | 255 | 216 | 0.222 | 32 | 0.235 | 104 | 0.234 | 80 | 0.143 |
|  |  | GA |  | 187 | 123 |  | 25 |  | 59 |  | 39 |  |
|  |  | GG |  | 36 | 30 |  | 9 |  | 9 |  | 12 |  |
|  |  | GA/GG^e^ |  | 223 | 153 | 0.132 | 34 | 0.458 | 68 | 0.107 | 51 | 0.116 |
|  |  | GA/AA |  | 442 | 339 |  | 57 |  | 163 |  | 119 |  |
|  |  | GG^f^ |  | 36 | 30 | 0.747 | 9 | 0.091 | 9 | 0.308 | 12 | 0.540 |
| 17 | *MT1M* | rs9936741 | 99.5 |  |  |  |  |  |  |  |  |  |
|  |  | TT |  | 367 | 310 | **0.038** | 57 | 0.211 | 141 | 0.337 | 112 | 0.093 |
|  |  | CT |  | 102 | 58 |  | 8 |  | 30 |  | 20 |  |
|  |  | CC |  | 7 | 2 |  | 1 |  | 1 |  | 0 |  |
|  |  | CT/CC^e^ |  | 109 | 60 | **0.016** | 9 | 0.087 | 31 | 0.183 | 20 | 0.054 |
|  |  | CT/TT |  | 469 | 368 |  | 65 |  | 171 |  | 132 |  |
|  |  | CC^f^ |  | 7 | 2 | 0.332 | 1 | 0.978 | 1 | 0.688 | 0 | 0.356 |
| PCH: pregnancy with chronic hypertension; GH: gestational hypertension; PE/PESCH: preeclampsia, or preeclampsia superimposed on chronic hypertension.  ^a^Two-side chi-square test for genotype distribution between HDP and controls.  ^b^Two-side chi-square test for genotype distribution between PCH and controls.  ^c^Two-side chi-square test for genotype distribution between GH and controls.  ^d^Two-side chi-square test for genotype distribution between PE/PESCH and controls. | | | | | | | | | | | | |
